# P060. Vitamin D deficiency in episodic migraine, chronic migraine and medication-overuse headache patients

**DOI:** 10.1186/1129-2377-16-S1-A184

**Published:** 2015-09-28

**Authors:** Rosario Iannacchero, Amerigo Costa, Aida Squillace, Luca Gallelli, Umberto Cannistrà, Giovambattista De Sarro

**Affiliations:** Center for Headache and Adaptive Disorders, Unit of Neurology, Department of Neuroscience and Sense Organs, Azienda Ospedaliera “Pugliese-Ciaccio”, Catanzaro, Italy; Unit of Clinical Pharmacology and Pharmacovigilance, Department of Health Science, Magna Graecia University, Catanzaro, Italy

## Background

Various studies have hypothesized a common inflammatory pathogenesis for headache and hypovitaminosis D (HD), showing that migraine patients have low levels of vitamin D [1]. Other studies did not confirm this [2]. Recently, a relationship between HD and reduced pharmacological response has been hypothesized, while vitamin D supplementary intake in HD patients may contribute to clinical improvement [3]. Aim of our prospective study was to evaluate the relationship between vitamin D levels and headache frequency and management in a cohort of patients accessing to our Centre for Headache from April 2015 to October 2015. We present preliminary data about patients enrolled until June 2015.

## Materials and methods

We enrolled 22 patients (6 males, 16 females; 45.41±11.22 mean age) accessing our Center from April 2015 to June 2015. We assigned patients to one of two groups according to headache frequency: Group A patients had a clinical history of episodic migraine (EM; n=7; < 8 headache days/month); Group B patients had a clinical history of chronic migraine and/or medication-overuse headache (CH/MOH; n=15; > 8 headache days/month). We excluded < 18 and > 55 year old patients and patients already supplementing vitamin D. At access, all patients received neurological and headache assessment, pharmacological evaluation, disability assessment and psychological evaluation. We took blood samples to obtain the dosage of vitamin D (normal values = 30-100 ng/ml). Using SOFA Statistics 1.4.4 software, we calculated descriptive indicators and Pearson's correlation coefficient (r) between headache frequency and vitamin D levels. We compared groups on vitamin D levels using independent samples Student's t-test. We set p < 0.05 as threshold of statistical significance. The local ethical committee approved the study design. All patients signed an informed consent to the research.

## Results

**Figure 1:**
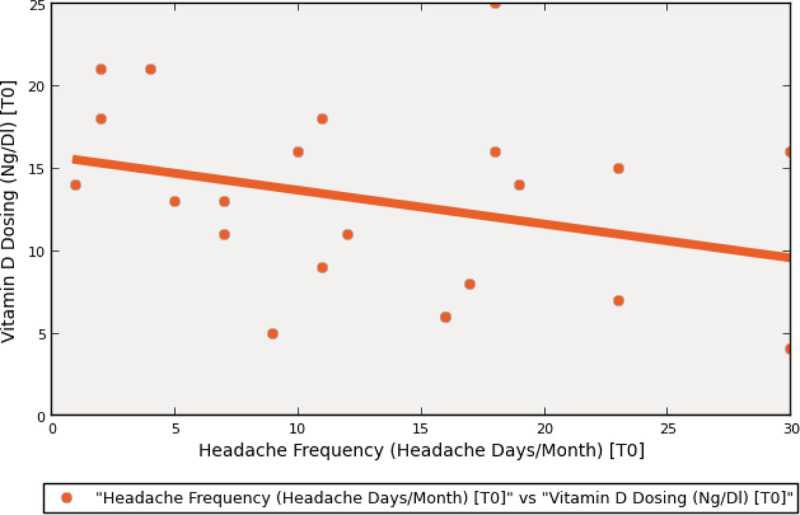
Scatter plot between headache frequency and vitamin D levels.

Vitamin D levels (M±DS) in all patients (n=22) fell below the normal range (13.05±5.70). Vitamin D levels were lower (M±DS) among Group A (CM+MOH) patients (11.73±5.98) than Group B (EM) patients (15.86±4.10); Student's t-test showed no statistically significant difference between groups on vitamin D levels (t = 1.642; p = 0.116). While Pearson's test did not show correlation between vitamin D levels and headache frequency (r = -0.308; p = 0.163), scatterplot suggests that such hypothetical inverse relationship, given a sample expansion, should be investigated (fig. 1).

## Conclusions

Migraine patients appear to have a high rate of hypovitaminosis D. Since vitamin D may coadiuvate the absorption of medication, further studies should investigate HD role in reducing migraine pharmacological management efficacy.

Written informed consent to publish was obtained from the patient(s).
